# Platelet-Rich Plasma Accelerates the Return to Sport in Athletes with Acute Muscle Injuries: A Systematic Review and Statistical Fragility Index-Based Meta-Analysis of Randomized Controlled Trials

**DOI:** 10.1186/s40798-026-01017-w

**Published:** 2026-05-12

**Authors:** Alessandro Bensa, Gae Fattini Fellini, Angelo Boffa, Alberto Grassi, Kristian Samuelsson, Giuseppe Filardo

**Affiliations:** 1 Service of Orthopaedics and Traumatology, Department of Surgery, EOC, Lugano, Switzerland; 2 Università della Svizzera Italiana, Faculty of Biomedical Sciences, Lugano, Switzerland; 3 Dipartimento di Scienze Biomediche e Neuromotorie, Alma Mater Studiorum Università di Bologna, Bologna, Italy; 4 Clinica Ortopedica e Traumatologica 2, IRCCS Istituto Ortopedico Rizzoli, Bologna, Italy; 5 Department of Orthopaedics, Institute of Clinical Sciences, The Sahlgrenska Academy, University of Gothenburg, Gothenburg, Sweden

**Keywords:** Muscle, PRP, Platelet-rich plasma, Athletes, Return to sport

## Abstract

**Background:**

Muscle lesions are common sport injuries resulting in prolonged absence from sport activity and long rehabilitation periods. The aim of this systematic review and meta-analysis was to quantify the clinical benefits of platelet-rich plasma (PRP) injections in treating acute muscle injuries.

**Methods:**

The search was conducted on PubMed, Cochrane Library, and Web of Science in January 2026. The PRISMA guidelines were used. Inclusion criteria were: randomized controlled trials (RCTs) published in English comparing PRP injections with control (rehabilitation or placebo) for the treatment of acute muscle injuries. The outcomes analysed were: time to return to sport (RTS), Visual Analogue Scale (VAS) for pain, re-injury and complication rates. Two sub-analyses were performed, one on the double-blind RCTs and one on the RCTs focusing on hamstrings. A fragility analysis was performed using the fragility index (FI) and the continuous FI (CFI). The quality of each article was assessed using the Cochrane RoB 2 and the GRADE tools.

**Results:**

Among the 4969 articles retrieved, nine RCTs (474 patients) were included. PRP provided faster RTS both in the overall analysis (*p* < 0.001, mean difference (MD) = 7.5, CFI = 188) and in the two sub-analyses (*p* < 0.001, MD = 8.8, CFI = 67 and *p* = 0.001, MD = 7.5, CFI = 97 respectively), as well as superior VAS improvement in the hamstring sub-analysis (*p* = 0.006, MD = 0.4, CFI = 22). No difference was found in terms of re-injury and complication rates between the two groups (FI = 5 and FI = 7, respectively). The evaluation using the RoB 2 tool showed that four studies had a “low risk” of bias and five had a “high risk” of bias. The GRADE evaluation showed a limited quality of evidence of the analysed outcomes.

**Conclusion:**

PRP produced a faster RTS compared to controls in acute muscle injury patients, both in the overall analysis and in the sub-analyses. PRP was able to produce a statistically higher pain relief only in the hamstring subgroup, while no difference was found in terms of re-injury and complication rates, suggesting a similar safety profile when compared to rehabilitation alone and placebo. The fragility analysis supported the benefits in terms of RTS, although the high-quality literature addressing this topic remains limited, warranting caution in the interpretation of the current results.

## Key Points

- PRP injections can accelerate return to sport in acute muscle injury patients.
- PRP injections can produce superior pain relief to control in hamstring lesions.
- PRP injections do not increase re-injury and complication rates compared to control in acute muscle injury patients.

## Background

Muscle lesions are among the most common injuries in sport [1], representing approximately a third of all injuries and causing more than a quarter of the total injury-related absences from sport [2]. In more than 90% of the cases, the four major muscle groups of the lower limb are affected, including primarily the hamstrings, followed by adductors, quadriceps, and calf muscles [3]. Such injuries can have considerable repercussions on both players and their teams, resulting in prolonged absence from sport and long rehabilitation periods [3, 4]. Moreover, re-injury rates have been reported to be as high as 43% in different sports, with hamstring musculo-tendinous relapses reaching even 50% [5]. Treatment goals are therefore to restore the pre-injury functional and activity levels while minimizing the time to return to sport (RTS) and the recurrence risk [6].

Many therapeutic approaches are currently used to address these types of injuries, including protection, rest, ice, compression, and elevation, followed by rehabilitative exercises and gradual training therapy to recondition the injured structure, as well as other available options like anti-inflammatory medications, electrotherapeutic modalities, hyperbaric oxygen therapies, photothermal therapy, or injection strategies such as prolotherapy [7–9]. Considering the limits of the available options, new orthobiologic approaches are generating increasing interest due to their potential to accelerate the muscle healing process [10]. Among these, platelet-rich plasma (PRP) showed to reduce inflammatory distress and to stimulate anabolism in different tissues, exploiting the high concentration of growth factors, cytokines, and bioactive molecules stored in platelet α-granules, which may play an important role in muscle regeneration and myogenesis [11]. Preclinical evidence suggested that autologous PRP injections might promote the muscle recovery process and reduce recovery time, prompting their application also in the clinical setting [11, 12]. However, the high-level clinical evidence on the use of PRP for the management of acute muscle injuries presented conflicting results. Previous attempts to analyse the literature are based on limited data, and the use of this orthobiologic product to promote the healing process and RTS remains controversial [13].

The aim of this systematic review and meta-analysis was to quantify the clinical effectiveness of PRP injections in improving the outcome of acute muscle injuries in terms of time of RTS, as well as pain improvement, re-injuries, and complications.

## Methods

### Data Source, Search, and Study Selection

The study was registered on the international prospective register of systematic reviews (PROSPERO registration number CRD42024545885). The Preferred Reporting Items for Systematic Reviews and Meta-analysis (PRISMA) guidelines were used [14]. The following databases were systematically searched on January 6, 2026, with no time limitation and without any filters: PubMed, Cochrane Library, and Web of Science using the following string: (PRP OR platelet-rich plasma OR plasma rich in growth factors OR platelet derived growth factor OR platelet derived OR platelet gel OR platelet concentrate OR PRF OR platelet rich fibrin OR ACP OR autologous conditioned plasma OR PRGF OR platelet lysate) AND (muscle injury). Duplicates were removed and, subsequently, all records were checked for eligibility by titles and abstracts. The full-text article was read when not enough information could be retrieved from the abstract. The following inclusion criteria were used: randomized controlled trials (RCTs) published in English comparing PRP injections with control (either rehabilitation or placebo) for the treatment of acute muscle injuries. Exclusion criteria were: non-RCTs, biomechanical studies, in vitro studies, review articles, case reports, letters to the editor, and editorials. When two or more papers evaluated the same patient cohorts, the relevant data were extracted from each study, but only the study with a longer follow-up was considered in order to avoid data duplication. The article selection process was independently performed by two authors (A.Be., G.F.F.) with disagreement on study eligibility resolved by a third author (A.Bo.).

### Data Extraction and Outcome Measurement

Patient demographic details, including sex, age, body mass index, type of muscle injury, and level of activity, were extracted. Details of study design, such as level of evidence, type of randomization, blinding of patients or outcome assessors, injection protocol, PRP characteristics, and rehabilitation or placebo details, were collected. For the outcome measurements, time to RTS, Visual Analogue Scale (VAS) for pain improvement from pre- to post-treatment values, re-injury and complication rates, strength, range of motion (ROM) or flexibility, functional scores, and details of imaging evaluation were extracted as well. Two authors independently extracted the trial information using a standardized extraction form (A.Be., G.F.F.) with disagreement resolved by a third author (A.Bo.).

### Assessment of Risk of Bias and Quality of Evidence

The risk of bias and quality of evidence of each article was assessed independently by two authors (A.Be. and G.F.F.), with disagreements resolved by consensus with a third author (A.Bo.). For the risk of bias, the Cochrane risk-of-bias tool for randomized trials Version 2 (RoB 2) was used [15]. RoB 2 is structured into a fixed set of domains of bias, focusing on different aspects of trial design, conduct, and reporting. Within each domain, a series of questions (“signaling questions”) aims to elicit information about features of the trial that are relevant to the risk of bias. A proposed judgement on the risk of bias for each domain is generated by an algorithm, based on answers to the signaling questions. Judgement can be “Low” or “High” risk of bias or can express “Some concerns”. For each plotted outcome of the comparative meta-analysis, the quality of evidence was evaluated according to the Grading of Recommendations Assessment, Development and Evaluation (GRADE) guidelines. In the GRADE system, the baseline rating of RCTs is considered “high”. Five criteria are used to downgrade one or two steps in cases of “serious” or “very serious concerns”: risk of bias in individual studies, inconsistency of results between studies, indirectness of evidence, imprecision, and publication bias. The overall quality of evidence can be graded as “high”, “moderate”, “low”, or “very low”.

### Statistical Analysis

The statistical analysis and the Forest plotting were carried out according to Neyeloff et al. using the Meta XL tool for Microsoft Excel by an independent professional statistician [16]. The analysis was carried out using random effects (DerSimonian & Laird) for the weighted mean difference (MD) of continuous variables and Peto method for the odds ratio (OR) of dichotomous variables [17]. Heterogeneity was evaluated using the I² statistical test. The presence of significant heterogeneity was considered with I^2^ ≥ 25% [18]. When no heterogeneity was found with I^2^ < 25%, a fixed effect model was used to estimate the expected values and 95% confidence intervals, otherwise a random-effect model was applied and an I^2^ metric was evaluated for the random effect to check the correction of heterogeneity. A p value of 0.05 was considered significant. Two sub-analyses were performed, one on the double-blind RCTs and the other on the RCTs focusing on hamstrings. A fragility analysis was performed using the fragility index (FI) for categorical variables according to the method previously described by Atal et al. [19] and the continuous FI (CFI) for continuous variables according to the method described by Caldwell et al. [20]. Additionally, the fragility quotient (FQ) was calculated as the absolute FI number divided by the total sample size, to provide a “relative” measure of fragility accounting for the sample size [21]. The FI represents the minimum number of patients from one or more trials included in the meta-analysis for whom an event status modification (i.e., changing an event to a non-event or a non-event to an event) would change the pooled treatment effect from statistically significant to non-significant or vice versa. The calculation method is based on an iterative process to re-evaluate the statistical significance of the pooled treatment effect of modified meta-analyses, iteratively derived from the original meta-analysis by performing single event-status modifications in each arm of each trial in turn. The FI was calculated for the dichotomous variables available (i.e. yes/no outcomes), namely re-injury and complication rates. The CFI represents the same concept as the FI applicable to continuous variables. The CFI is determined by an iterative algorithm [20] taking as input two datasets representing a continuous variable and performing the following steps: (1) Conduct a Welch *t*-test. (2) If the p value is < 0.05, identify the dataset with the higher mean and move the data point closest to (but still greater than) the mean of that set to the other set. (3) Repeat the Welch *t*-test. (4) If the p value remains < 0.05, repeat step 2. Otherwise, the CFI is reported as the number of iterations necessary to achieve a non-significant p value. The CFI was calculated for continuous variables, namely RTS and VAS improvement, having statistically significant results. The interpretation of the FI and FQ values obtained was based on cut-offs previously reported by Xing et al. and describing six categories (Table 1): extremely fragile, moderately fragile, slightly fragile, slightly robust, moderately robust, and extremely robust [21]. All statistical analyses were carried out by an independent professional statistician with Microsoft Excel 2010.

**Table 1:** Cut-offs for fragility analysis interpretation

Category	Significant to non-significant		Non-significant to significant	
	FI	FQ	FI	FQ
Extremely fragile	≤ 1	≤ 0.01	≤ 1	≤ 0.01
Moderately fragile	1–3	0.01–0.02	2–4	0.01–0.03
Slightly fragile	2–5	0.02–0.03	3–5	0.02–0.05
Slightly robust	6–14	0.03–0.08	6–7	0.06–0.10
Moderately robust	10–26	0.04–0.16	7–12	0.08–0.16
Extremely robust	≥ 27	≥ 0.16	≥ 13	≥ 0.17

*FI* fragility index,* FQ* fragility quotient

## Results

### Risk of Bias and Quality of Evidence

**Fig. 1:**
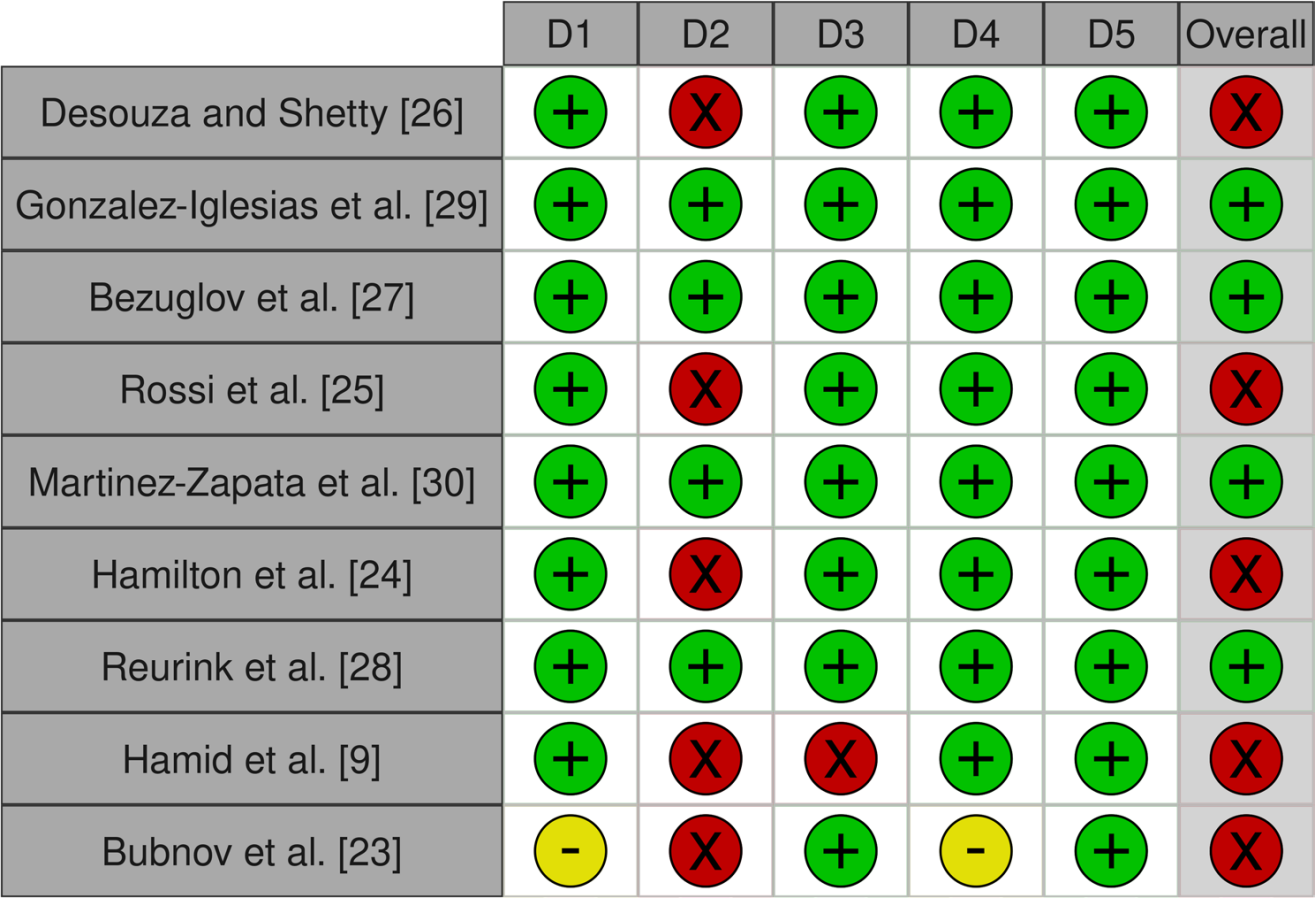
Cochrane risk of bias tool for randomized trials Version 2 (RoB 2) - green = low risk, yellow = some concerns, red = high risk; D1: bias arising from the randomization process, D2: bias due to deviations from intended intervention, D3: bias due to missing outcome data, D4: bias in measurement of the outcome, D5: bias in selection of the reported result

The evaluation using the RoB 2 tool showed that four studies had a “low risk” of bias and five had a “high risk” of bias. A summary of the risk of bias assessment of the included RCTs is illustrated in Fig. 1 [22]. The GRADE evaluation showed that the level of evidence of the results was moderate for two outcomes, low for five outcomes and very low for one outcome. A summary of the quality of evidence assessment of the meta-analysed outcomes is illustrated in Table 2.

**Table 2:** GRADE evaluation

Outcomes	Risk of bias	Inconsistency	Indirectness	Imprecision	Publication bias	Other	Quality of the evidence
RTS overall	Serious	Serious	No	No	No	No	Low
RTS double-blind	No	No	No	Serious	No	No	Moderate
RTS hamstring	Serious	No	No	Serious	No	No	Low
VAS overall	Serious	Serious	No	Serious	No	No	Very low
VAS double-blind	No	No	No	Serious	No	No	Moderate
VAS hamstring	Serious	No	No	Serious	No	No	Low
Re-injury	Serious	No	No	Serious	No	No	Low
Complications	Serious	No	No	Serious	No	No	Low

### Characteristics of the Included Studies and Patients

**Fig. 2:**
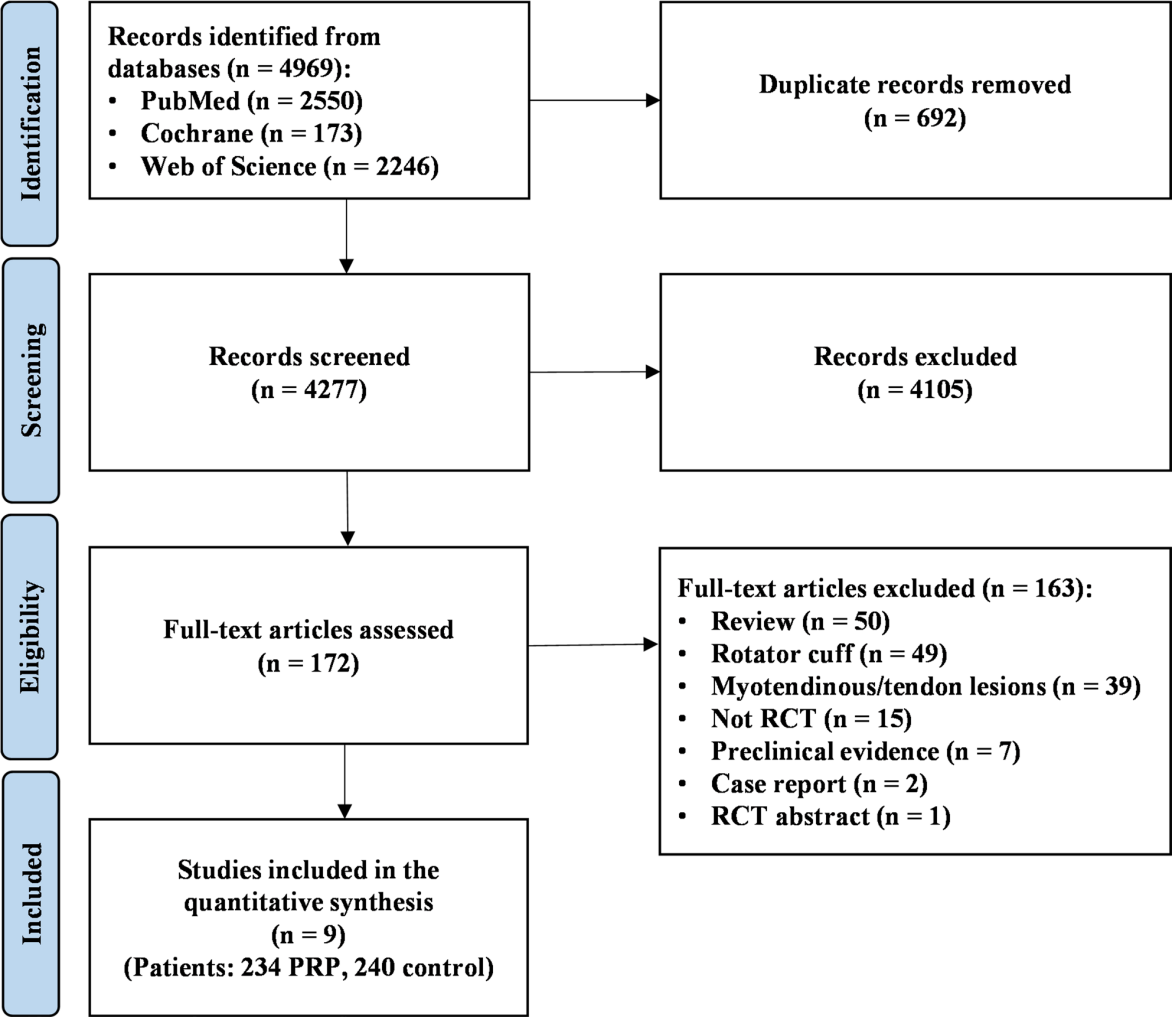
PRISMA (Preferred Reporting Items for Systematic Reviews and Meta-Analysis) flow diagram.* PRP* platelet-rich plasma

Out of 4969 records retrieved, a total of nine RCTs were included in the quantitative data synthesis (Fig. 2). The total number of patients in the included studies was 504 and a total of 474 patients were included in the meta-analysis comparing PRP and control: 234 patients in the PRP group (93.1% men, 6.9% women, mean age 27.2 ± 7.9 years) and 240 in the control group (92.1% men, 7.9% women, mean age 27.2 ± 7.8 years). Of these patients, 458 (96.1%) completed the last follow-up and were included in the meta-analysis, 227 in the PRP group and 231 in the control group. In the five single-blinded studies [9, 23–26], PRP was compared to rehabilitation alone. The remaining four studies were double-blinded and compared PRP to saline in two studies [27, 28], to Traumeel (a homeopathic preparation) in one study [29], and to hematoma evacuation alone in one study [30]. The patients and treatment characteristics of the included studies are reported in Table 3. Six studies [9, 24, 26–29] exclusively included hamstring injuries, while the remaining three [23, 25, 30] included several injury locations such as hamstrings, rectus femoris, quadriceps, gastrocnemius, thigh, foot and ankle, and shoulder. Three studies reported the lesion length and width [24, 25, 30], two studies reported only the lesion length [9, 28], and four studies did not report the lesion dimensions [23, 26, 27, 29]. The number of PRP injections ranged from one to three, with six studies [9, 23–25, 27, 30] detailing a single-injection protocol, two studies [28, 29] performing two injections, and one study performing either two or three injections according to muscle injury grade [26]. In all studies, except one [24], PRP injections were performed under ultrasound (US) guidance. The platelet concentration, as reported by the authors of the included articles, ranged from 700000/mL to 1381 ± 430 × 10^9^/L, the leukocyte concentration from 0.02 × 10^9^/L to 38.3 × 10^9^/L, and the volume injected from 2.5 to 8 mL. Three studies activated PRP using calcium chloride (CaCl_2_) [27, 29, 30], one using calcium gluconate (C₆H₁₁O₇)₂Ca [26], three studies stated they did not utilize any activating agent [9, 24, 25], while two studies did not report details on the method of activation [23, 28]. The PRP and lesion characteristics of the included studies are reported in Table 4. Only one study had an additional treatment group other than PRP or control [24]: the 30 patients in this group received a platelet-poor plasma (PPP) injection.

The outcomes extracted included: time to RTS (nine studies), complication rate (nine studies), re-injuries rate (seven studies), VAS pain (six studies), strength (four studies), imaging healing parameters (four studies), ROM or flexibility (two studies), Hamstring Outcome Score and Satisfaction (one study). The meta-analysis was performed on RTS, VAS pain, re-injury, and complication rates.

**Table 3:** Patients and treatment characteristics of the included studies

Study	Blinding	Patients included		Patients follow-up		Sex PRP		Sex Control		Age		Type of control	Diagnosis
		PRP	Control	PRP	Control	Male	Female	Male	Female	PRP	Control		
Desouza and Shetty [26]	Single	30	30	30	30	NR	NR	NR	NR	24.5 ± 3.8	25.1 ± 4.1	Physiotherapy	US and MRI
Gonzalez-Iglesias et al. [29]	Double	21	20	20	20	19	1	20	0	25.3 ± 4.4	24.8 ± 5.1	Traumeel	US
Bezuglov et al. [27]	Double	20	20	20	20	20	0	20	0	27 ± 3.3		Saline + physiotherapy	US and MRI
Rossi et al. [25]	Single	35	40	34	38	27	8	31	9	22.9 ± 3.5	21.8 ± 3.2	Physiotherapy	US
Martinez-Zapata et al. [30]	Double	28	32	27	30	NR	NR	NR	NR	45.9 ± 10.3	45.3 ± 9.8	Hematoma evacuation alone	US
Hamilton et al. [24]	Single	30	30	28	27	30	0	30	0	26.6 ± 5.9	25.5 ± 5.7	Physiotherapy	MRI
Reurink et al. [28]	Double	41	39	41	39	39	2	37	2	28.0 ± 7.0	30.0 ± 8.0	Saline	MRI
Hamid et al. [9]	Single	14	14	12	12	13	1	11	3	20.0 ± 6.5	21.0 ± 5.1	Physiotherapy	US
Bubnov et al. [23]	Single	15	15	15	15	15	0	15	0	24.0	24.0	Physiotherapy	US

*MRI* magnetic resonance imaging,* PRP* platelet-rich plasma,* US* ultrasound

**Table 4:** PRP and lesion characteristics of the included studies

Study	Platelet concentration	Leukocyte concentration	PRP volume injected (mL)	Number of injections	PRP activation	Lesion site		Lesion width (cm)		Lesion length (cm)	
						PRP	Control	PRP	Control	PRP	Control
Desouza and Shetty [26]	NR	NR	2.5-3	2–3	(C₆H₁₁O₇)₂Ca	Hamstrings	Hamstrings	NR	NR	NR	NR
Gonzalez-Iglesias et al. [29]	400–500 × 10^9/L	0.02 × 10^9/L	6.1 ± 1.2 5.6 ± 0.8	2	CaCl_2_	Hamstrings	Hamstrings	NR	NR	NR	NR
Bezuglov et al. [27]	0.7 × 10^9/L	NR	8	1	CaCl_2_	Hamstrings	Hamstrings	NR	NR	NR	NR
Rossi et al. [25]	NR	NR	Proportional to the injury	1	No	Hamstrings (16), quadriceps (7), gastrocnemius (12)	Hamstrings (18), quadriceps (8), gastrocnemius (11)	1.6 ± 0.6	1.7 ± 0.5	2.3 ± 1.0	24.5 ± 8.7
Martinez-Zapata et al. [30]	1381 ± 430 × 10^9/L	0.11 ± 0.06 × 10^9/L	4–8	1	CaCl_2_	Gastrocnemius (32), rectus femoralis (1)	Gastrocnemius (36), rectus femoralis (2)	6.1 ± 6.3	7.5 ± 9.7	11.4 ± 5.7	13.0 ± 14.6
Hamilton et al. [24]	765.8 ± 423.6 × 10^9/L	26.1 ± 13.7 × 10^9/L	3	1	No	Hamstrings	Hamstrings	2.4 ± 1.3	2.3 ± 1.3	15.8 ± 8.2	15.5 ± 6.1
Reurink et al. [28]	433 ± 128 × 10^9/L	1.9 ± 2.1 × 10^9/L	3	2	NR	Hamstrings	Hamstrings	NR	NR	11.1 ± 6.0	12.7 ± 6.0
Hamid et al. [9]	1297 × 10^9/L	38.3 × 10^9/L	3	1	No	Hamstrings	Hamstrings	NR	NR	3.4 ± 1.1	2.3 ± 1.0
Bubnov et al. [23]	NR	NR	5	1	NR	Thigh (10), foot and ankle (5), shoulder (2)	Thigh (8), foot and ankle (5), shoulder (4)	NR	NR	NR	NR

*PRP* platelet-rich plasma

### Return to Sport

**Fig. 3:**
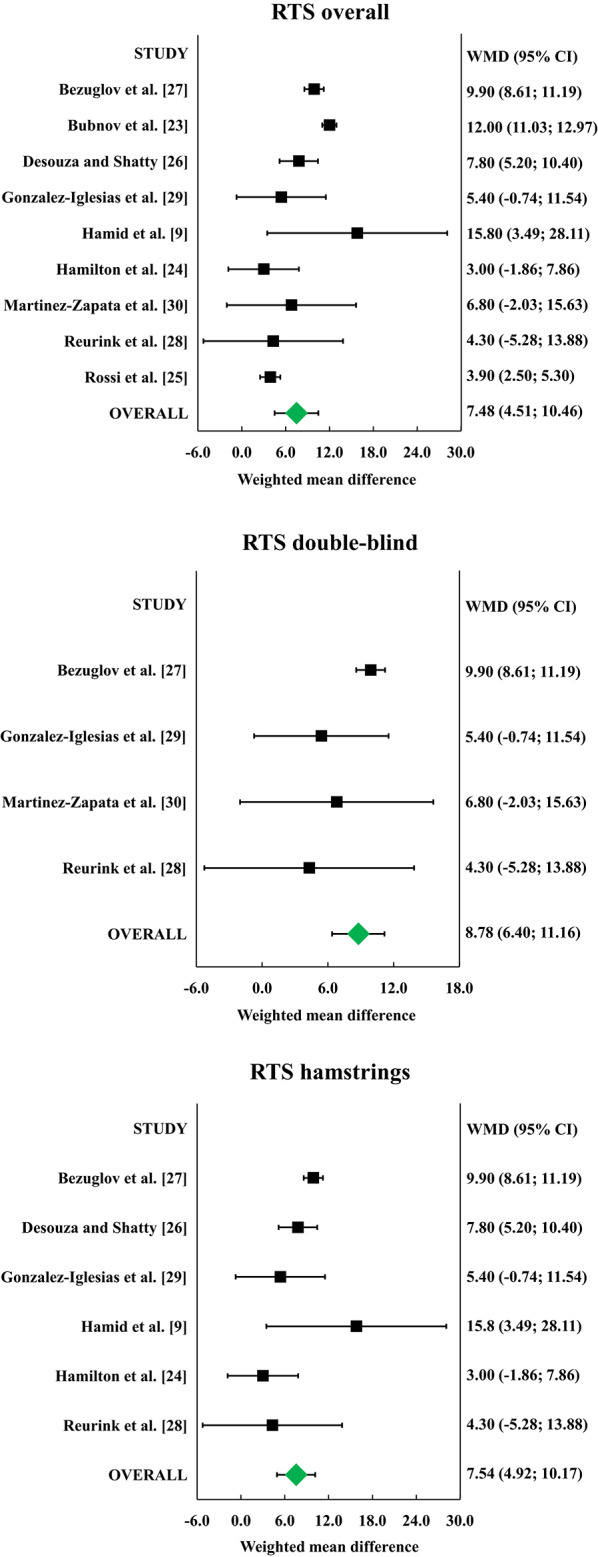
Forest plots of the individual studies and pooled Weighted Mean Difference (WMD) for time to return to sport (RTS), including a 95% confidence interval. Platelet-rich plasma (PRP) provided shorter RTS in the overall analysis as well as in the sub-analyses of double-blind studies and of studies focusing on hamstrings. Green: statistically significant

All the nine included RCTs reported the time to RTS in the PRP and control groups. The meta-analysis of RTS (Fig. 3) including 458 patients showed a statistically significant difference in favour of the PRP group compared to the control group of 7.5 days (*p* < 0.001, I^2^ = 92%). The CFI was 188, meaning that 188 patients should be moved from one group to the other in order to reverse the statistical significance of the meta-analysis. The sub-analysis considering the four double-blind RCTs including 217 patients confirmed a statistically significant difference in favour of the PRP group of 8.8 days (*p* < 0.001, I^2^ = 16%), with a CFI of 67. The sub-analysis considering the six RCTs focusing on hamstrings and including 299 patients showed a statistically significant difference in favour of the PRP group of 7.5 days (*p* = 0.001, I^2^ = 58%), with a CFI of 97.

Specific factors influencing the RTS could not be meta-analysed. The study published by Gonzalez-Iglesias et al. [29] reported that the anatomical location of the injury was identified as the strongest predictor of the return to play. Participants with myofascial or intramuscular injuries were able to return to sport after 25.7 days, which was 7.9 ± 5.2 days sooner than the patients with myotendinous injuries. The subgroup analyses performed in the study by Desouza et al. [26] showed that PRP treatment consistently resulted in a faster RTS compared to the control group across all subcategories of muscle injury severity as well as across different hamstring injury sites. In the study by Hamilton et al. [24], PRP was able to produce a quicker RTS compared to PPP, with a statistically significant difference of 5.7 days.

### VAS Improvement

**Fig. 4:**
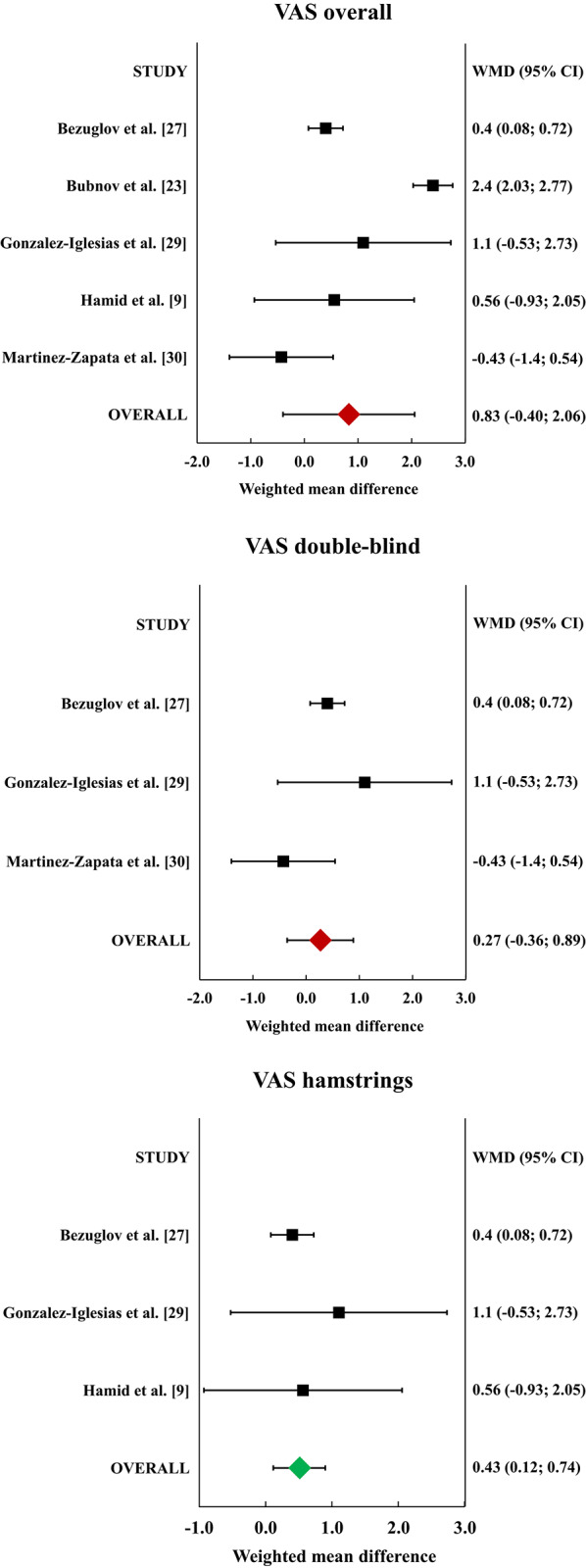
Forest plots of the individual studies and pooled Weighted Mean Difference (WMD) for Visual Analogue Scale (VAS) pain improvement, including a 95% confidence interval. No statistically significant difference was detected between platelet-rich plasma (PRP) and controls in the overall analysis and in the sub-analysis of double-blind studies. PRP provided superior VAS improvement in the sub-analysis of studies focusing on hamstrings. Green: statistically significant, red: not statistically significant

Five of the included RCTs reported either VAS improvement or VAS pre-treatment and post-treatment values [9, 23, 27, 29, 30]. The meta-analysis of VAS improvement (Fig. 4) including 279 patients did not show a statistically significant difference between the PRP and the control groups (I^2^ = 95%). The sub-analysis considering the three double-blind RCTs including 137 patients did not show a statistically significant difference between the two groups (I^2^ = 41%). The sub-analysis considering the three RCTs focusing on hamstrings and including 189 patients showed a statistically significant difference in favour of the PRP group of 0.4 points (*p* = 0.006, I^2^ = 6%), with a CFI of 22.

### Re-Injury and Complications

**Fig. 5:**
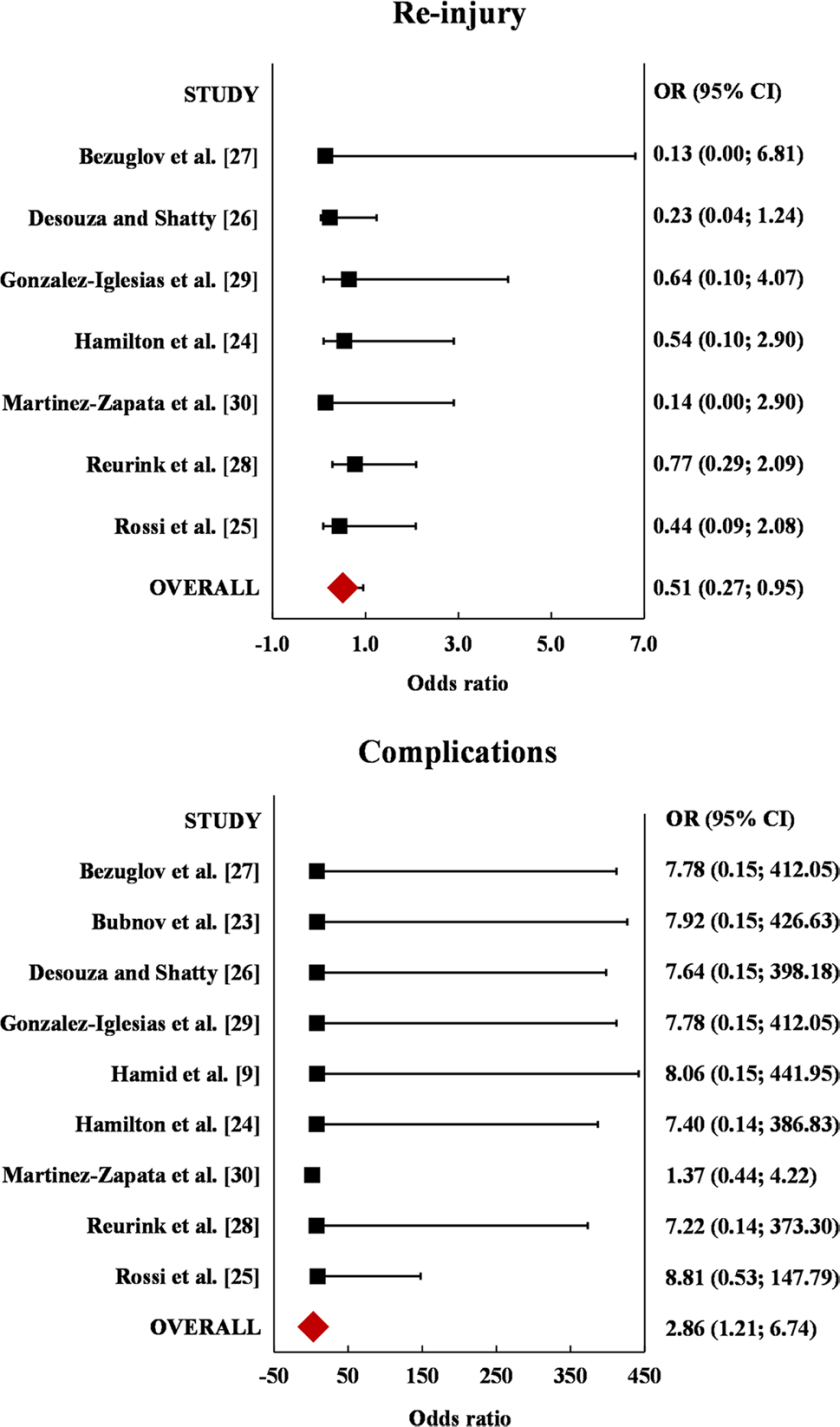
Forest plots of the individual studies and odds ratios for re-injury rate and complication rates, including a 95% confidence interval. In both cases, no statistically significant difference was detected between platelet-rich plasma (PRP) and control. Red: not statistically significant

Seven of the included RCTs reported the re-injury rate [24, 25, 27–30] and all nine reported the complication rate (Fig. 5). The meta-analysis of the re-injury rate including 398 patients did not show a statistically significant difference between the PRP and the control groups (I^2^ = 0%). The FI for the re-injury rate meta-analysis was 5, meaning that a status change of 5 patients would be necessary to reverse the non-significant meta-analysis to a significant one, with a FQ of 0.013. The meta-analysis of the complication rate including 458 patients did not show a statistically significant difference between the PRP and the control groups (I^2^ = 0%). The FI for the complication rate meta-analysis was 7, meaning that a status change of 7 patients would be necessary to reverse the non-significant meta-analysis to a significant one, with a FQ of 0.015. FI results can be classified as “slightly fragile” for the re-injury rate and “moderately robust” for the complication rate. FQ results can be classified as “moderately robust” for both the re-injury rate and the complication rate.

### Other Outcomes

#### Strength

Four studies evaluated muscle strength and reported no differences between the PRP and the control groups at the final follow-up [9, 23, 24, 28]. Only Bubnov et al. [23] observed higher strength in PRP-treated patients, but only during the first 2 weeks.

#### Imaging

The two studies that evaluated ultrasonographic appearance found no difference in muscle healing between PRP and control [23, 30]. Regarding muscle oedema reduction at MRI, two studies showed no difference between the two groups [24, 28], while one study reported a statistically significant difference in favour of PRP at day 21.

#### ROM or Flexibility

Among the two studies evaluating ROM or flexibility, Reurink et al. [28] reported no differences, while Bubnov et al. [23] reported a higher ROM in patients treated with PRP at all the evaluated follow-ups.

#### Functional Scores

Reurink et al. [28] used a subjective score (Hamstring Outcome Score and Satisfaction) to assess muscle function and found no significant differences between patients treated with PRP or isotonic saline injection.

## Discussion

The main finding of this meta-analysis is that PRP injections accelerate RTS in patients with muscle lesions. These results have been confirmed in the sub-analysis on studies focusing specifically on hamstrings, where PRP also showed a superior pain relief albeit of limited clinical relevance. No difference was found in terms of re-injury and complication rates.

PRP has gained attention as a promising therapeutic approach in the musculoskeletal field because of its biological properties and regenerative potential. Among the different applications of this orthobiologic treatment, its use for the management of acute muscle injuries showed promising results at preclinical level, supporting its testing in the clinical setting [31]. Previous smaller systematic reviews and meta-analyses tried to quantify the benefits of PRP for the treatment of this condition, showing conflicting results reported by the very limited number of high-level clinical trials addressing this topic [32, 33]. Particularly, the largest meta-analysis published so far concluded that the limited and low-quality evidence provided by the available literature was not sufficient to provide robust justifications for the use of PRP to treat acute muscle injuries, showing no relevant benefits in terms of RTS, re-injuries, and complications compared to control [13]. The present meta-analysis builds upon these findings and expands the previously performed analyses, including a higher number of studies and of outcomes while adding a statistical fragility index-based analysis to evaluate the robustness of the obtained results.

Among the aims of acute muscle injury treatments, RTS represents one of the highest priorities for both players and teams, as well as one of the most relevant parameters to evaluate the effectiveness of the applied therapeutic approach [34]. While suggesting promising results of PRP in accelerating RTS after acute muscle injuries, previous studies failed to provide decisive conclusions supporting this product due to the limited amount of available evidence. The present meta-analysis explored a larger body of literature and showed that PRP was able to shorten the time to RTS by more than a week compared to controls. This represents a difference of decisive clinical relevance, especially for professional players and elite teams, with considerable impact on the athlete’s physical and mental condition as well as with potential economic and career implications [35]. Moreover, both the sub-analyses on double-blind RCTs and on studies focusing on hamstrings obtained analogous results, confirming this finding in the highest-levels trials included while showing its specific validity in the muscle group most frequently affected by this pathology. The fragility analysis showed a good level of robustness, supporting the validity both of the main analysis and of the sub-analyses performed. Of note, one of the included RCTs showed that PRP was able to accelerate RTS compared to PPP [24]. Despite some authors suggesting positive results of PPP for the treatment of acute muscle injuries based on an extremely limited number of patients in low quality studies [36, 37], this RCT represents the strongest evidence produced so far on the topic, supporting the superiority of PRP over PPP in the management of this pathology.

Beyond the need to provide a quick RTS, another priority of acute muscle injury management remains the prevention of re-injuries and complications. The present meta-analysis showed no statistically significant difference between PRP and controls in terms of both re-injury and complication rates, with the fragility analysis showing the overall robustness of the meta-analyses on these outcomes. These results confirm the findings of previous smaller literature analyses by including a larger number of studies, suggesting a similar safety profile of PRP when compared to rehabilitation alone and placebo [13, 31, 38]. Despite the FI suggesting slightly fragile results for the re-injury analysis and moderately robust results for the complications analysis, the FQ showed a moderate robustness for both results when accounting for the number of patients respectively included in these analyses.

Another relevant outcome contributing to the overall results of acute muscle injury treatment is represented by pain improvement. Previous systematic reviews addressed this aspect reporting inconclusive results due to the limited amount of data available, which also prevented from the possibility to meta-analyse this parameter [13, 31, 32]. The present study was able to expand these findings by performing a meta-analysis of VAS improvement from pre- to post-treatment values. The results did not show significant differences between PRP and control in terms of pain relief, with only a difference of limited clinical relevance in favour of PRP in the hamstring subgroup. However, it is worth noticing that the amount of studies reporting VAS results was limited and that a larger number of high-quality trials evaluating a superior number of patients are needed to further investigate this aspect and provide more conclusive results on the potential role of PRP in pain mitigation for this condition.

The overall encouraging results of PRP provided by the present meta-analysis should be interpreted with caution in light of the relatively small number of studies and therefore of patients included as well as because of their overall limited quality. In fact, a considerable variability in terms of risk of bias among the selected RCTs was detected by the RoB 2 analysis and the GRADE evaluation showed a low quality of evidence in the majority of the outcomes analysed. This is an aspect that should not be disregarded, since a direct correlation between low meta-analysis quality and positive results was previously demonstrated in the literature [39]. Nevertheless, the sub-analysis of the double-blind RCTs confirmed the results of the main analysis both in terms of RTS and of VAS pain score, representing a decisive difference from previous meta-analyses where the inconsistency between the overall analysis and the sub-analysis of the highest-level trials considerably weakened the reliability of the obtained results [13]. Another relevant aspect complicating the results interpretation is the considerable heterogeneity of PRP formulations among the included studies, which reflects the overall complexity in the field of PRP injections. Numerous aspects can vary across different protocols, including blood volume harvested, use of anticoagulant, number and speed of centrifugations, final volume obtained, leukocyte content, overall number of platelets, their integrity and activation method, the possibility of cryopreserving them or using fresh products, and the variability in application modalities, with single injections or injection cycles and different volumes and platelet concentrations [40–43]. In this context, the RCTs included in the present meta-analysis presented a considerable variability across the aspects reported, including platelet and leukocyte concentration, volume injected, number of injections, as well as associated procedures (e.g. hematoma aspiration) and US guidance. Moreover, the heterogeneity in terms of lesion size and location across the studies further complicates the results interpretation. In this scenario, future studies should aim at investigating the factors influencing the effectiveness of PRP injections and the treatment response based on the lesion characteristic in order to provide patients and clinicians with the best possible indications to maximize their benefits and ultimately optimize the management of acute muscle injuries in clinical practice.

This systematic review and meta-analysis presents some limitations that require consideration. First, the limited number of studies and patients included in the meta-analysis impacts the overall quality of evidence and may possibly cause some of the outcomes not to reach statistical significance in the event of a true positive effect. However, the complex study design, with both randomization and possibly multiple blinding, makes it difficult to perform such studies in a context different from high-volume centres and/or in a multi-centric fashion. Second, the selected RCTs lacked standardization in data collection and reporting of outcome measures and associated follow-up timeframes, reducing the amount of data available for the meta-analysis. Additionally, the limited number of studies addressing strength, ROM or flexibility, and muscle imaging as well as the different methods used to investigate these outcomes did not allow to include them in the meta-analysis. The available RCTs also presented substantial variability in terms of characteristics and posology of the injected PRP, reflecting the considerable heterogeneity of the field of PRP injections. Moreover, the variability of lesion size and location across the included studies did not allow for specific sub-analyses, except for the hamstring muscle group. Finally, the presence of risk of bias and the overall limited quality of evidence in the analysed outcomes warrants caution in the interpretation of the findings reported.

Despite these limitations, the present systematic review and statistical fragility index-based meta-analysis provided a thorough investigation of the available literature, showing important results on the use of PRP injections for the treatment of acute muscle injuries. PRP showed encouraging results in terms of RTS compared to controls while exhibiting a similar safety profile in terms of both re-injury and complication rates. More high-level clinical trials are needed to strengthen these findings and to identify standardized protocols able to optimize PRP administration, as well as the identification of lesion characteristics that could obtain the greatest benefit from this treatment. As demonstrated for other PRP applications in the musculoskeletal system, PRP has a large placebo effect and more studies with appropriate blinding are needed to further elucidate its real potential in comparison to placebo [44, 45]. Future studies should ensure accurate randomization and blinding, precise description of PRP characteristics, and homogeneous populations as well as reporting of the results. Moreover, imaging evaluations of the tissue repair and clinical outcomes should be documented both in the early stages, which are crucial for athletes aiming for a quick RTS, as well as in the long-term, to investigate whether tissue modifications may reduce the recurrence of muscle injury.

## Conclusion

PRP produced a faster RTS compared to controls in acute muscle injury patients, both in the overall analysis and in the sub-analyses. PRP was able to produce a statistically higher pain relief only in the hamstring subgroup, while no difference was found in terms of re-injury and complication rates, suggesting a similar safety profile when compared to rehabilitation alone and placebo. The fragility analysis supported the benefits in terms of RTS, although the high-quality literature addressing this topic remains limited, warranting caution in the interpretation of the current results.

## Data Availability

The datasets used and/or analysed during the current study are available from the corresponding author on reasonable request.

## Abbreviations

ACP: Autologous conditioned plasma
CaCl_2_: Calcium chloride
CFI: Continuous fragility index
FI: Fragility index
FQ: Fragility quotient
GRADE: Grading of Recommendations Assessment, Development and Evaluation
MD: Mean difference
MRI: Magnetic resonance imaging
OR: Odds ratio
PRF: Platelet rich fibrin
PRGF: Plasma-rich in growth-factors
PRISMA: Preferred Reporting Items for Systematic Reviews and Meta-analysis
PROSPERO: International prospective register of systematic reviews
PRP: Platelet-rich plasma
RCTs: Randomized controlled trials
RoB 2: Cochrane risk of bias tool for randomized trials Version 2
ROM: Range of motion
RTS: Return to sport
VAS: Visual Analogue Scale
US: Ultrasound
WMD: Weighted mean difference
